# Association between IL-10 gene polymorphisms (− 1082 A/G, -819 T/C, -592 A/C) and hepatocellular carcinoma: a meta-analysis and trial sequential analysis

**DOI:** 10.1186/s12885-023-11323-1

**Published:** 2023-09-08

**Authors:** Teresa Tan Yen Mei, Htar Htar Aung, Wong Siew Tung, Cho Naing

**Affiliations:** 1 School of Medicine, International Medical University, Kuala Lumpur, Malaysia; 2 School of Medicine, University of Adelaide, Adelaide, Australia; 3 Faculty of Tropical Health and Medicine, James Cook University, Queensland, Australia

**Keywords:** IL-10 gene polymorphisms, Hepatocellular carcinoma, Genetic association, meta-analysis, Trial sequential analysis

## Abstract

**Background:**

The carcinogenesis of hepatocellular carcinoma is complicated, and genetic factor may have the role in the malignant transformation of liver cells. IL-10 gene polymorphisms have been investigated for their potential roles in hepatocellular carcinoma This study aimed to investigate the relationship between polymorphisms of IL-10 (-1082 A/G, -819 T/C, -592 A/C), and hepatocellular carcinoma by performing a meta-analysis with eligible individual studies.

**Methods:**

This study followed the PRISMA 2020 Checklist. Relevant studies were searched in health-related databases. The Newcastle-Ottawa Scale criteria were used to evaluate the studies quality. Pooled odds ratio (OR) and its 95% confidence interval (CI) were used to determine the strength of association between each polymorphism and hepatocellular carcinoma using five genetic models. Stratification was done by ethnic groups. Trial sequential analysis (TSA) was performed to determine the required information size.

**Results:**

Fifteen case-control studies (n = 8182) were identified. Overall, the heterozygous model showed a marginal significant association only between IL-10 (-1082 A/G) and hepatocellular carcinoma risk (OR: 0.82, 95% CI: 0.67-1.00, 9 studies). On stratification, IL-10 (-1082 A/G) was significantly associated with hepatocellular carcinoma risk in the non-Asian population under dominant (OR: 0.62, 95% CI: 0.45–0.86, 4 studies), heterozygous (OR: 0.60, 95% CI: 0.43–0.85) and allelic models (OR: 0.79, 95% CI: 0.64–0.99). IL-10 (-819 T/C) was significantly associated with hepatocellular carcinoma risk only among non-Asians under the dominant (OR: 1.47, 95% CI: 1.02–2.13, 8 studies), recessive (OR: 1.99, 95% CI: 1.03–3.86, and homozygous models (OR: 2.18, 95% CI: 1.13–4.23). For IL-10 (-592 A/C) with 11 studies, there was no significant association with hepatocellular carcinoma in all five genetic models (*P* values > 0.5). TSA plots indicated that the information size for firm evidence of effect was sufficient only for the analysis of IL-10 (-592 A/C), but not for the − 1082 A/G or -819 T/C.

**Conclusions:**

Findings suggest that IL-10 (-1082 A/G and − 819 T/C) polymorphisms are associated with hepatocellular carcinoma in ethnic-specific manner. However, this evidence is not conclusive because the sample size was insufficient. IL-10 (-592 A/C) polymorphism was not associated with hepatocellular carcinoma albeit with sufficient information size. Future well-designed large case-control studies on IL-10 (-1082 A/G and − 819 T/C) with different ethnicities are recommended.

**Supplementary Information:**

The online version contains supplementary material available at 10.1186/s12885-023-11323-1.

## Background

Hepatocellular carcinoma is the most common primary liver cancer, accounting 70–80% of total primary liver cancers [1]. Worldwide, it is the sixth most common type of cancer [2], and the third leading cause of cancer related deaths in 2020 [3, 4], albeit with variation in sex and geographic distribution. According to the GLOBOCAN estimate in 2020, the age-standardised incidences were highest in Eastern Asia, followed by South-Eastern Asia and Northern Africa [3], with male predominance in most countries [5–8].

Due to nonspecific symptoms at the early stages, the mortality of hepatocellular carcinoma is very high [9], and the mean survival time for end-stage hepatocellular carcinoma patients is around four to six months [10]. Numerous studies reported risk factors for the development of hepatocellular carcinoma, including chronic hepatitis B virus (HBV) and hepatitis C (HCV) infections [11], aflatoxin-contaminated food consumption [12], and alcohol consumption [4, 11] As a matter of fact, the carcinogenesis of hepatocellular carcinoma is complicated, and both epigenetic and genetic factors may have their roles in the malignant transformation of liver cells [10]. Since hepatocellular carcinoma is attributed to prolonged inflammation of liver cells, inflammatory markers like cytokines may have the roles in hepatocellular carcinoma development [13].

Among the genetic factors, Interleukin-10 (IL-10) is one of the anti-inflammatory cytokines [14] as well as a multifunctional cytokine, which can inhibit development of tumour and disease progression. Studies have reported that a lack of IL-10 may stimulate the secretion of pro-inflammatory cytokines that inhibit anti-tumor immune responses and enhance growth of tumor [15–17]. However, the exact mechanisms of these polymorphism in cancer development and growth is not fully understood [18]. Moreover, polymorphisms of various genes control and alter the cytokines production (IL-10 in this case), and individual variations exit [19]. Hence, it is crucial to understand the role of particular single nucleotide polymorphisms (SNPs). Although chronic HBV and HCV infections are the common risk factors of hepatocellular carcinoma, only a few chronic cases with HBV and HCV develop hepatocellular carcinoma later in their lives [9]. Therefore, it has hypothesized that host genetic factors may play a part in malignant transformation of liver cells [10]. Understanding of IL-10 genetic polymorphisms may help to estimate the influence of genetic alteration on the development of hepatocellular carcinoma.

There is a surge of individual studies that investigated the roles of IL-10 in patients with hepatocellular carcinoma. These studies varied in racial decent of participants, sample sizes, and the quality of study design, and these could contribute to heterogenous findings. Meta-analysis is a method that combines results from data collected from all eligible studies. There are published meta-analyses that assessed IL-10 on hepatocellular carcinoma [20, 21]. However, these published reviews did not provide evidence on adequate information size, and hence the results were inconclusive. As(single nucleotide polymorphisms (SNPs) in the cytokine genes are known to affect cytokine production levels, we focused on three SNPs of IL-10 (-1082 A/G, -819 T/C, -592 A/C) in this study. Taken together, the objective was to investigate the relationship between polymorphisms of IL-10 (-1082 A/G, -819 T/C, -592 A/C), and hepatocellular carcinoma by performing a meta-analysis.

## Methods

We conducted the current study in adherence to the PRISMA 2020 checklist for reporting our meta-analysis (Additional File 1). A protocol of this study was approved by the Ethics Review Committee of the International Medical University in Malaysia (ID: BMS I/2021(10)). This study only used data from published studies. The need for consent from participants was waived by the Ethics Review Committee of the International Medical University in Malaysia.

### Search strategy

Relevant studies were searched in electronic databases of PubMed, Ovid Medline, Cochrane library, EBSCOHOST, Science Direct, Latin American and Caribbean Health Sciences Literature (LILACS), and Google scholar. Keywords and MeSH terms were used with Boolean operators: [“Interleukin-10” or “IL-10”] AND [“polymorphism” or “gene polymorphisms”] AND [“hepatocellular carcinoma” or “liver cancer”]. To capture any additional studies, we performed a snowball method of manual cross-referencing of the retrieved studies and relevant systematic reviews. The search was restricted to studies published in English until June 2022. Search strategies are provided in Additional File 2.

### Inclusion and exclusion criteria

Human studies that assessed hepatocellular carcinoma were included, if they.

i) assessed IL-10 gene polymorphisms, − 1082 A/G (rs1800870; rs 1,800,896), -819 T/C (rs1800871; rs 3,021,097), and/or -592 A/C (rs1800872);
ii) conducted case-control or nested case-control studies, irrespective of the method of DNA analysis;
iii) compared hepatocellular carcinoma patients with the controls (healthy controls or non-hepatocellular carcinoma participants);
iv) provided sufficient information to extract genotype frequencies in both cases and controls; and.
v) measured the outcome with odds ratio (OR) along with 95% confidence intervals (CI) (at least, provided enough data to estimate these).

We assessed whether the distribution of genotypes in the control group of the studies included was consistent with Hardy-Weinberg equilibrium (HWE) [22].

Hepatocellular carcinoma in this analysis was as defined in the primary studies. When more than one publication used the same participants, a publication with more comprehensive information was considered.

Studies that did not fit to the inclusion criteria (e.g., not case-control study, not genetic association studies, genetic studies with no genotype frequency) were not considered. Studies that assessed treatment response, disease severity, drug efficacy, and pre-clinical studies were excluded.

### Data extraction

The two investigators (TYM and HHA) independently extracted information from each study using a piloted data extraction sheet. Information collected were: first author, publication year, study country, number of cases/controls, age group, male%, polymorphism frequencies in cases/controls, the racial descent (Asian or non-Asian), and minor allele frequency (MAF) in the controls, and HWE status (if MAF and HWE were not provided, we calculated it). Any discrepancy between the two investigators was resolved by discussion with the third investigator (CN).

### Study quality assessment

The two investigators (TYM and HHA) independently evaluated the methodological quality of eligible studies using the Newcastle–Ottawa scale (NOS). The NOS checklist covers three main domains (selection, exposure, comparability) in eight items, and each item was awarded 1 or 2 stars in maximum for high quality, and a final score obtained was between 0 and 9 stars [23]. We considered studies with ≥ 7 stars as high quality. Any variations between the two investigators were settled through a discussion with the third investigator (WST).

### Statistical analysis

The genotype frequencies in the control were checked for consistency using the HWE, and the exact test for goodness-of-fit was applied (*p* > 0.05) [24]. Described elsewhere [25], for individual studies, the strength of the association between IL-10 (-1082 A/G as an example) and hepatocellular carcinoma was estimated using OR and its 95%CI. For pooling of the estimates across studies, we calculated summary ORs and corresponding 95% Cis. We used random-effect model (Der Simonian and Laird method), accounting statistical heterogeneity of the studies. Otherwise, fixed-effect model (the Mental-Haenszel method) was used. Heterogeneity was evaluated with the *I*^2^ statistics (the percentage of total variation across studies), reflecting the heterogeneity rather than chance. *I*^2^ values greater than 50% is regarded as a substantial heterogeneity [26]. We calculated the pooled ORs and its 95% CIs under five genetic models (i.e., dominant, recessive, homozygous, heterozygous, and allelic models). The generic formula used for these calculations [27] is presented in Additional File 3.

To detect the sources of heterogeneity, we planned to perform meta-regression with covariates of personal factors (e.g. age, gender), and environmental factors (e.g. alcohol consumption, smoking, HBsAg status). Due to paucity or inconsistent reporting of these data, it was not able to do a meta-regression.

In small studies, a statistically significant finding would be actually a false-positive report probability (FPRP) [28]. We performed the FPRP test with the use of a pre-set FPRP < 0.2 and assigned prior probabilities of 0.25, 0.1, 0.01 or 0,001 to examine an OR of 1.5 (or its reciprocal 0.67 = 1/1.5 for ORs less than 1) of associated with the hepatocellular carcinoma risk. To evaluate whether an association is “noteworthy”, we used a FPRP cut-off value of 0.2. Statistical power and FPRP were computed by the Excel spreadsheet provided elsewhere [28].

To investigate the stability of results, we performed a sensitivity analysis with leave-one-out meta-analysis by removal of one study at a time. We planned to assess the publication bias by visual inspection of funnel plots [29]. This was done only for the − 592 A/C, where a minimum of 10 studies were available for this assessment.

### Trial sequential analysis

Trial sequential analysis (TSA), an approach that adjusts for random error risk, was done for estimation of the required information size [30]. For dichotomous outcomes in this case, we calculated the information size adjusted for heterogeneity (diversity, D²) between trials (studies in this case) using the parameters described elsewhere [31]. Proportion of events in the control group estimated from the included studies (overall mean value), anticipated intervention effect (relative risk reduction) of 15%, alpha of 5% (one main outcome), and beta of 20% [32, 33]. It is classified as ‘potentially spurious evidence of effect’ (if the cumulative Z-curve did not cross the monitoring boundaries), or as ‘firm evidence of effect’ (if the cumulative Z-curve crossed the monitoring boundaries) [31].

Meta-analysis was done with Stata 16 (StataCorp TX), while TSA was with TSA version 0.9 beta (Copenhagen Trial Unit, Centre for Clinical Intervention Research, Copenhagen).

## Results

**Fig. 1:**
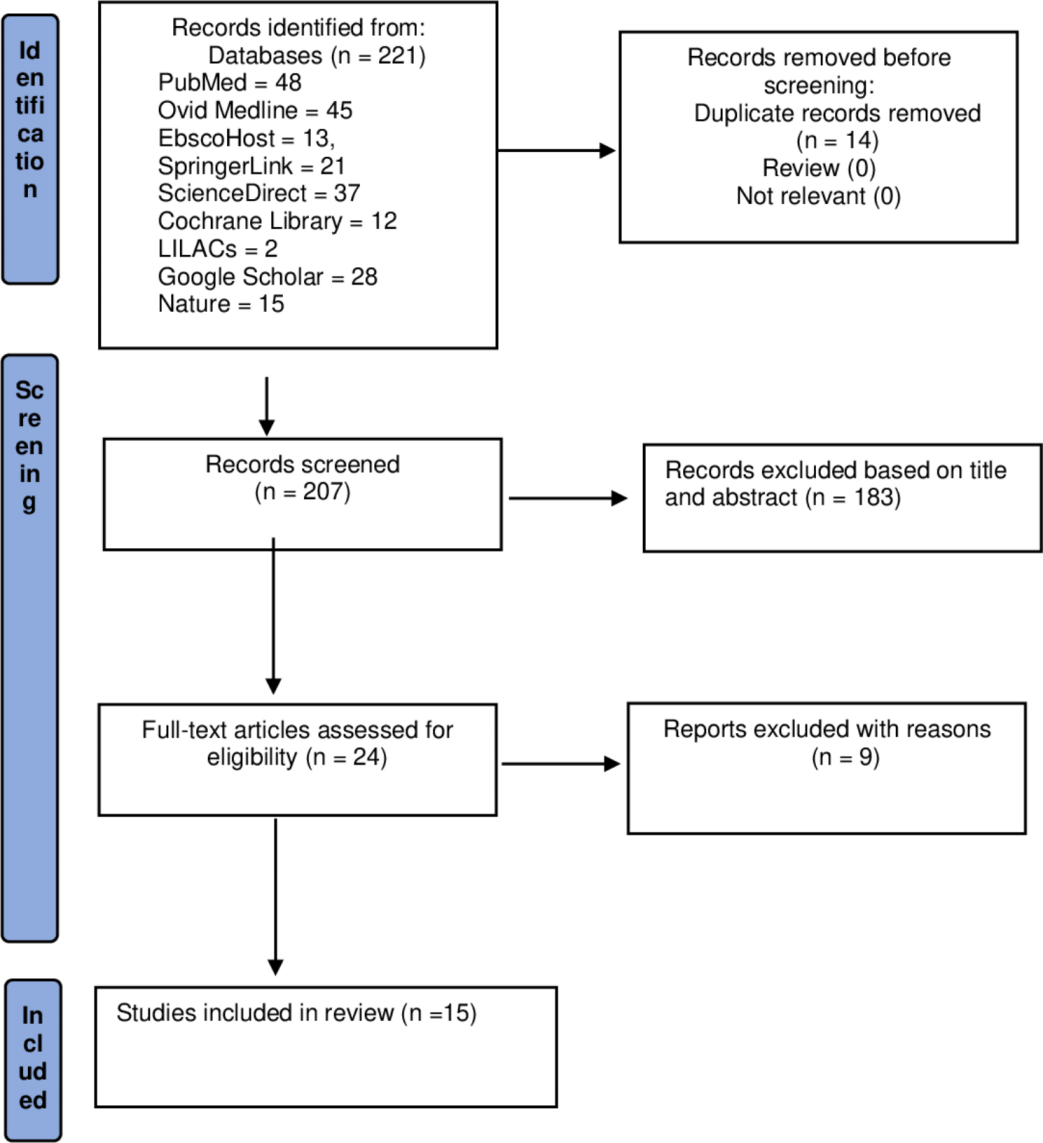
Study selection flowchart

Figure 1 shows a study selection process. Initially, 221 studies were yielded from the database searches. After removal of 14 duplicates, 207 studies were further screened for the title and abstract. Twenty-four full-text studies were evaluated for eligibility, and finally, a total of 15 studies were identified for this meta-analysis [32, 34–48]. The reasons for nine excluded studies were presented in Additional File 4.

### Study characteristics

**Fig. 2:**
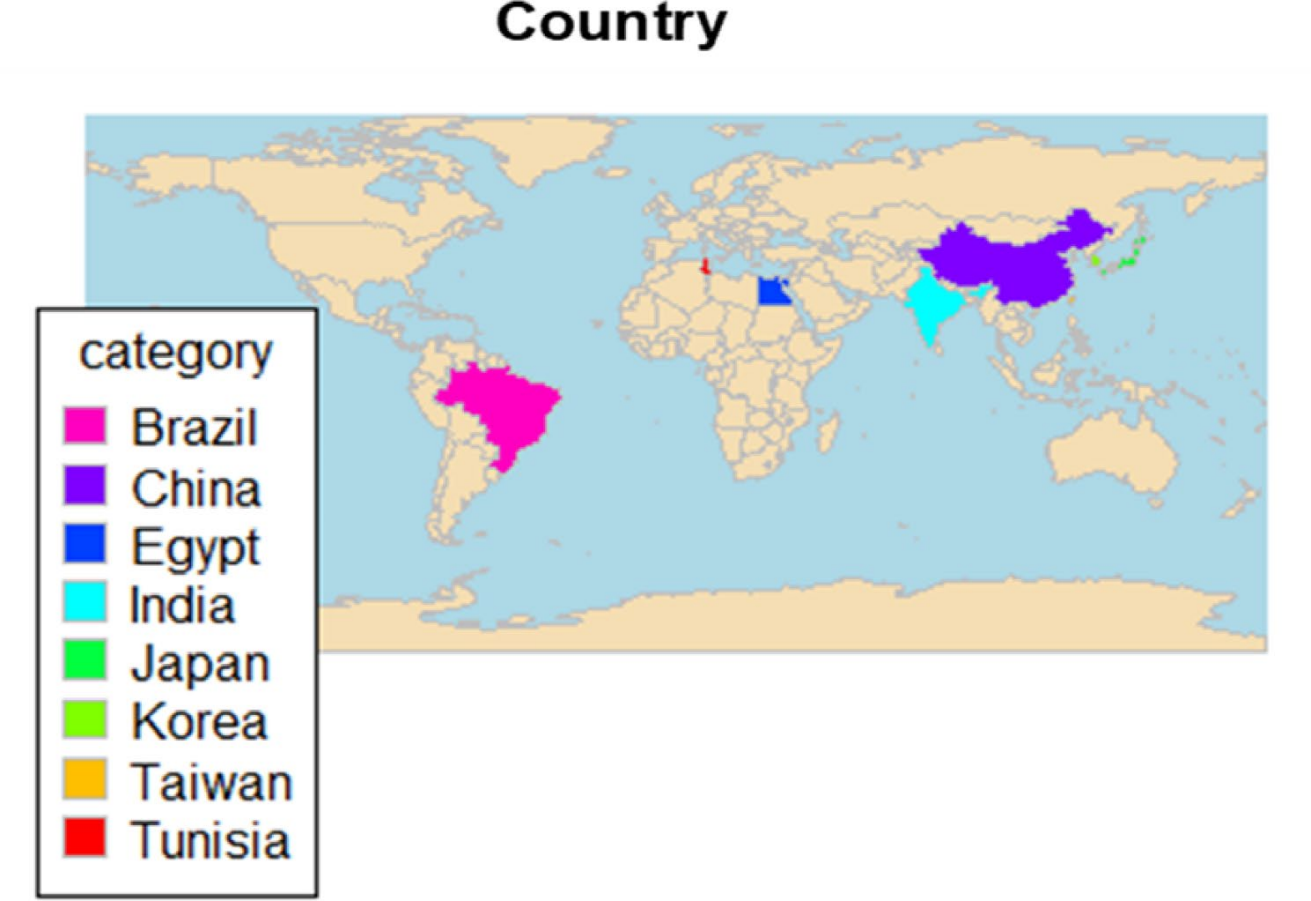
Geographic distribution of the included studies

Table 1. presents the main characteristics of the included studies. A total of 15 studies involving 8182 participants (2923 cases and 5259 controls) were identified. These studies were conducted across eight countries most frequently in China [37, 40, 42, 47, 48] (Fig. 2). Study samples varied from 100 [35] to 1504 participants [37], and the years of publication spanned from 2003 [40, 45] to 2020 [36, 39, 43]. With regard to ethnic groups, ten studies (67%) were done with the Asians population [36, 37, 40–42, 44–48], while five studies (33%) were with the non-Asian population [34, 35, 38, 39, 43]. Ten studies were hospital-based studies [34, 35, 37–40, 42, 43, 45–48], while 5 studies were done on patients at the outpatient clinic/special clinic [35, 36, 39, 43, 44]. Eight studies (53%) examined more than one SNP [32, 34, 40–42, 44, 45, 45], while nine [32, 34–36, 38–42, 45], eight [34, 36, 38, 40–44, 47], and 11 studies [32, 36, 37, 40–42, 44–48] examined the single SNP − 1082 A/G, -819 T/C, and − 592 A/C, respectively. Several genotyping methods were used in these studies, and the most frequently used genotyping methods were AS-PCR, TaqMan, and PCR-RFLP.

**Table 1:** Characteristics of study

Author, yr [ref]	Yr of publication	Country	Setting	Ethnic group	Samples (case/ control)	Age in yr^1^	Male%^1^	Underlying cause	Polymorphism	Genotyping method	HWE
Aroucha 2016 [34]	2016	Brazil	Hospital	Non-Asian	108/280	62.7 ± 8.7	68.5	HCV related HCC	-1082 A/G.-819 C/T, -592 A/C	TaqMan	Yes
Bahgat 2015 [35]	2015	Egypt	Community clinic	Non-Asian	50/50	52.30 ± 4.41	82	HCV related HCC	-1082 A/G	RT-PCR	Yes
Barooh 2020 [36]	2020	India	OPD	Asian	60/306	54.8 ± 9.1	49	HCV related HCC	-1082 A/G.-819 C/T, -592 A/C	PCR-RFLP	No
Bei 2014 [37]	2014	China	Hospital	Asian	720/784	48.65 ± 11.03	86	Newly diagnosed HCC	-592 A/C	TaqMan	Yes
Bouzgarrou 2009 [38]	2009	Tunisia	Hospital	Non-Asian	58/145	61.6 ± 9.8	34.5	HCV related HCC	-1082 A/G	AS- PCR-	Yes
El-Baky 2020 [39]	2020	Egypt	Hospital	Non-Asian	54/92	64.16 ± 12.1	90.7	HCV complicated with HCC	-1082 A/G	TaqMan	No
Heneghan 2003 [40]	2003	China	Hospital	Asian	98/175	55 year (range: 14–77 year)	92.9	HBV related HCC	-1082 A/G.-819 C/T, -592 A/C	PCR-SSCP	Yes
Migita 2003 [41]	2003	Japan	OPD	Asian	48/188	62.5 ± 8.9	81.25	HBV related HCC	-1082 A/G.-819 C/T, -592 A/C	PCR-SSP	No
Peng 2016 [42]	2016	China	Hospital	Asian	173/182	56.32 ± 7.55	36.42	HBV related HCC	-1082 A/G.-819 C/T, -592 A/C	PCR-RFLP	Yes
Saleh 2020 [43]	2020	Egypt	HCC clinic	Non-Asian	73/85	56.21 ± 4.62	58.9	HCV related HCC	-819 C/T	RT-PCR	No
Saxena 2014 [44]	2014	India	OPD	Asian	59/331	55.31 ± 12.67	18.66	HBV related HCC	-819 C/T, -592 A/C	AS-PCR	No
Shin 2003 [45]	2003	Korea	Hospital	Asian	230/792	Only cutoff age		HBV related HCC	-1082 A/G, -592 A/C	MAPA	No
Tseng 2006 [46]	2006	Taiwan	Hospital	Asian	208/528	55 (23–85)		HBV related HCC	-592 A/C	PCR/RFLP	Yes
Wang 2019 [47]	2019	China	Hospital	Asian	554/612 (277/306)	≤ 35- >50	84.8	HBV-related HCC^2^	-819 C/T, -592 A/C	PCR-based	No
Zhou 2017 [48]	2017	China	Hospital	Asian	430/709	< 55-≥55	88.1	HBV-related HCC	-592 A/C	MassARRAY	Yes

^1^: values for the cases; ^2^: 87%of the cases;

AS: Allele-specific; HCC: hepatocellular carcinoma; HWE: Hardy–Weinberg equilibrium: MAPA: multiplex automated primer extension analysis; OPD: Outpatient Department/Outpatient clinic; PCR: Polymerase chain reaction; PCR/RFLP: mis- matched PCR/restriction fragment length polymorphism; RT-PCR: Reverse transcription polymerase chain reaction; PCR-SSCP polymerase chain reaction-single-strand conformation polymorphism

Frequency of individual SNP is provided in Additional File 5. Seven studies (47%) did not follow HWE [36, 39, 41, 43–45, 47]. We retained them for an initial overall analysis.

### Methodological quality assessment

Based on the NOS criteria, the studies included achieved the scores between 5 and 9. Less than half of these studies (6/15, 40%) obtained the high scores (i.e., > 7.0 score) [36, 37, 40–42, 45] (Additional File 6).

### Genetic model assessments

#### IL-10 (-1082 A/G)

Overall, IL-10 **(**-1082 A/G) showed a tendency toward significant relationship with hepatocellular carcinoma under heterozygous model (OR: 0.82, 95%CI:0.67-1.0, *I*^*2*^ = 49%, fixed effect model, 9 studies), however other four genetic models did not (All *p* values > 0.05) (Table 2).

**Fig. 3:**
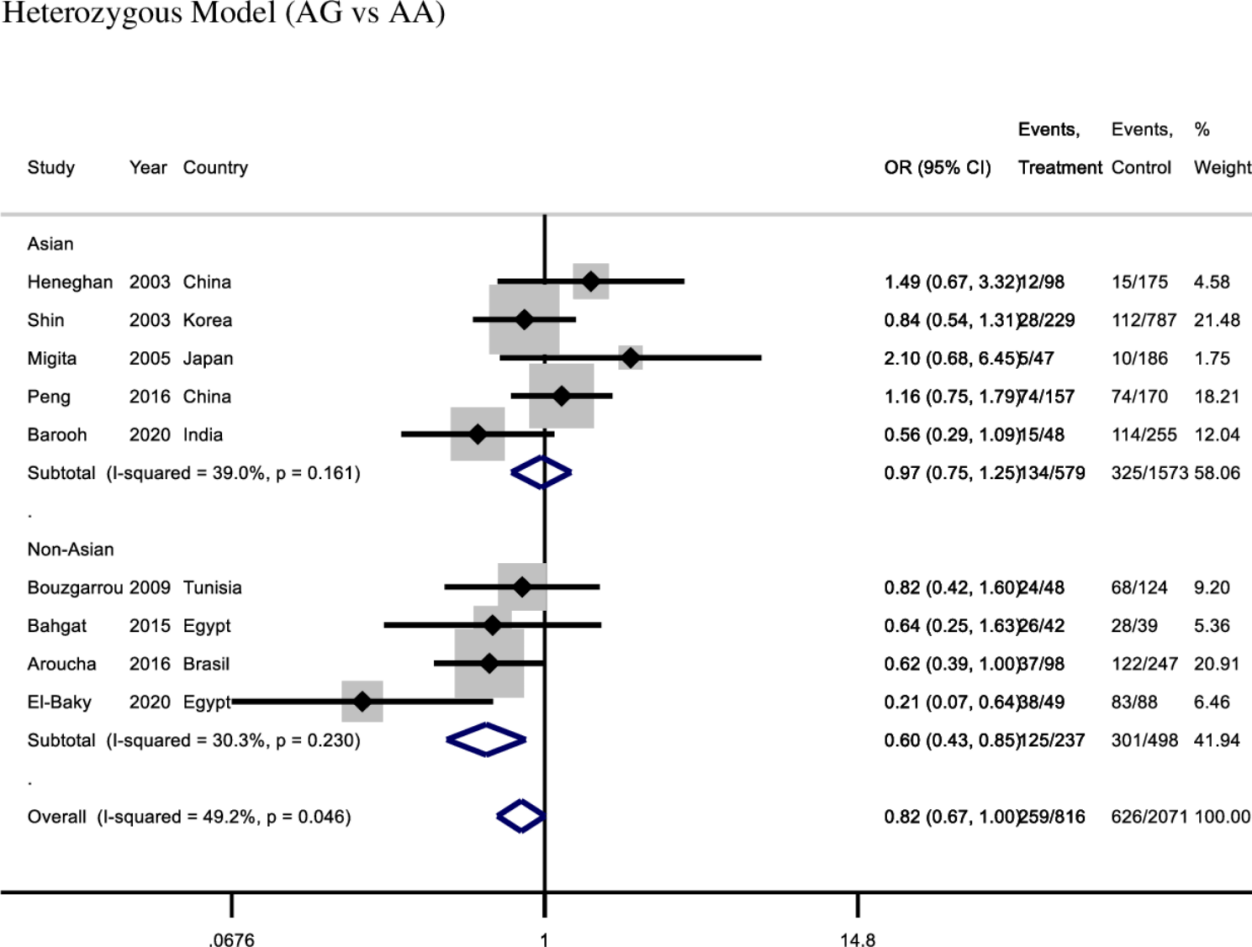
Forest plot of − 1082 A/ G under heterozygous model

For a subgroup of non-Asian population, there was a significant association between the IL-10 (-1082 A/G) and hepatocellular carcinoma in protection using the heterozygous model (OR: 0.60, 95% CI: 0.43–0.85, *I*^*2*^ = 30.3%, fixed effect model, four studies). However, for the Asian population, there was no significant association between the IL-10 (-1082 A/G) and hepatocellular carcinoma in heterozygous model (OR:0.97, 95% CI: 0.75–1.25, *I*^*2*^ = 39%, fixed effect model, five studies) (Fig. 3). There was a significant association between the IL-10 (-1082 A/G) and hepatocellular carcinoma in protection using the dominant model (OR: 0.62, 95% CI: 0.45–0.86, *I*^*2*^ *=* 33.1%, fixed effect model) and allelic model (G vs. A) (OR: 0.79, 95% CI: 0.64–0.99, *I*^*2*^ = 0.0%, fixed effect model) with the non-Asian population. However, there was no significant association with hepatocellular carcinoma under the remaining two genetic models (recessive and homozygous models). For a subgroup of Asian population (5 studies), there was no significant association with hepatocellular carcinoma under any of the five genetic models (Table 2).

**Table 2:** Associations with the risk of hepatocellular carcinoma

SNP	Number of studies included	Total participants	Genetic Model	Effect estimates, OR (95%CI); p value		
-1082 A/G	9 (5 Asian group, 4 non-Asian group)	3089		Overall	Asian group	Non-Asian group
			Dominant	0.85 (0.70–1.03) [p:0.09]	1.01 (0.79–1.29) [p:0.95]	**0.62 (0.45–0.86)** [p:0.004]
			Recessive	1.10 (0.79–1.54) [p:0.56]	1.30 (0.80–2.13) [p:0.29]	0.97 (0.62–1.52) [p:0.88]
			Heterozygous)	0**.82 (0.67–0.99)** [p:0.04]	0.97 (0.75–1.25) [p:0.65]	0**.60 (0.43–0.85)** [p:0.007]
			Homozygous	0.91 (0.64–1.30) [p:0.61]	1.20 (0.72–1.99) [p:0.65]	0.71 (0.43–1.17) [p:0.51]
			Allelic (	0.92 (0.80–1.07) [p:0.28]	1.05 (0.86–1.29) [p:0.63]	**0.80 (0.64–0.99)** [p:0.04]
-819 T/C	8 (6 Asian group, 2 non-Asian group)	3332		Overall	Asian group	Non-Asian group
			Dominant	1.25 (1.05–1.48) [p:0.37]	1.19 (0.98–1.45) [p:0.27]	**1.47 (1.02–2.13)** [p:0.032]
			Recessive	1.10 (0.81–1.50) [p:0.]	0.96 (0.74–1.26) [p:0.]	**1.99 (1.03–3.86)** [p:0.]
			Heterozygous	1.20 (1.00-1.43) [p:0.73]	1.19 (0.97–1.46) [p:0.49]	1.23 (0.82–1.84) [p:0.93]
			Homozygous	1.17 (0.80–1.72) [p:0.37]	0.97 (0.65–1.44) [p:0.12]	**2.18 (1.13–4.23**) [p:0.03]
			Allelic	1.08 (0.89–1.32) [p:0.35]	0.98 (0.81–1.19) [p:0.11]	1.59 (0.96–2.63) [p:0.64]
-592 A/C	11 (10 Asian group, 1 non-Asian group)	7516		Overall	Asian group	Non-Asian group
			Dominant	0.90 (0.68–1.18) [p:0.44]	0.86 (0.63–1.16) [p:0.32]	1.29 (0.82–2.03) [p:0.27]
			Recessive TT vs. CT	0.89 (0.72–1.11) [p:0.31]	0.86 (0.69–1.08) [p:0.2]	1.45 (0.75–2.78) [p:0.26]
			Heterozygous	0.96 (0.75–1.22) [p:0.31]	0.93 (0.71–1.21) [p:0.57]	1.21 (0.75–1.96) [p:0.43]
			Homozygous	0.93 (0.77–1.12) [p:0.43]	0.91 (0.75–1.10) [p:0.33]	1.32 (0.66–2.63) [p:0.44]
			Allelic	0.90 (0.76–1.08) [p:0.25]	0.87 (0.72–1.05) [p:0.15]	1.26 (0.91–1.74) [p:0.17]

Bold indicates significant at *p* value < 0.05. CI: Confidence interval; OR: Odds ratio:

#### IL-10 (-819 T/C)

Overall, Il-10 (-819 T/C) was associated with hepatocellular carcinoma susceptibility under dominant model (OR: 1.25, 95% CI:1.05–1.48, *I*^*2*^ = 15.7%, fixed effect model, 8 studies), and heterozygous model (OR: 1.20, 95% CI: 1.00-1.43, *I*^*2*^ = 0.0%, fixed effect model, 8 studies (Additional File 7). There was no significant association with hepatocellular carcinoma under other three genetic models (Table 2).

On a subgroup of the non-Asian population (two studies), There was significant association with hepatocellular carcinoma susceptibility under dominant model (OR: 1.47, 95% CI: 1.02–2.13, *I*^*2*^ *=* 0.0%, fixed effect model, two studies) and recessive model (OR:1.99, 95% CI: 1.03–3.86, *I*^*2 =*^ 46.1%, fixed effect model), and homozygous model (OR: 2.18, 95% CI: 1.13–4.23, *I*^*2*^ = 36.5%, fixed effect model, 111 participants) (Table 2). However, there was no significant association with hepatocellular carcinoma under the remaining two genetic models (heterozygous and allelic models). The subgroup Asian population (six studies) had no significant association with hepatocellular carcinoma under any five genetic models (Table 2).

#### IL-10 (-592 A/C)

Overall, there was no association with hepatocellular carcinoma under all five genetic models. On stratification by ethnic groups (Asian in 10 studies and non-Asian in one study), the Asian groups as well as the non-Asan group showed no significant association with hepatocellular carcinoma under any five genetic models (Table 2).

### FPRP test

Table 3 shows the FPRP of IL10 (-1082 A/G) and (-819 T/C) gene polymorphisms of significant association with hepatocellular carcinoma risk. At prior probability of 0.25 and 0.1 FPRP test results show that many associations of the non-Asian group remained “noteworthy”. At prior probability of 0.01 and 0.001, and statistical power to detect an OR of 1.5, none of the associations were considered “noteworthy” (FPRP < 0.2). This means at low level of prior probability of 0.001(1000: 1), none were “noteworthy” and may not be true association.

**Table 3:** False positive report probability power and value

Genetic model	OR (95%CI)	FPRP					
		P value	Statistical power	Prior probability			
				0.25	0.1	0.01	0.001
**-1082 A/G**							
**Dominant**							
Non-Asian	0.62 (0.45–0.86)	**0.004**	0.321	**0.038**	**0.105**	0.564	0.929
**Heterozygous)**							
Overall	0.82 (0.67–0.99)	**0.039**	0.982	**0.106**	0.263	0.797	0.975
Non-Asian	0.60 (0.43–0.85)	**0.004**	0.267	**0.043**	**0.12**	0.6	0.938
**Allelic**							
Non-Asian	0.80 (0.64–0.99)	**0.04**	0.953	**0.112**	0.275	0.806	0.977
**-819 T/C**							
**Dominant**							
Non-Asian	1.47 (1.02–2.13)	**0.042**	0.543	**0.188**	0.409	0.884	0.987
**Recessive**							
Non-Asian	1.99 (1.03–3.86)	**0.042**	0.202	0.383	0.651	0.954	0.995
**Homogenous**							
Non-Asian	2.18 (1.13–4.23)	**0.021**	**0.134**	0.321	0.587	0.940	0.994

FPRP: False positive report probability. Statistical power is the power to detect an odds ratio of 1.5 with the genetic variant (or, 0.67 = 1/1.5 for protective effect). The results in bold implies noteworthiness of association at 0.2 level by FPRP. Not performed FPRP test for IL10 (-592 A/C) as there was no significant association with hepatocellular carcinoma risk (See Table 2)

### Sensitivity analysis

**Fig. 4:**
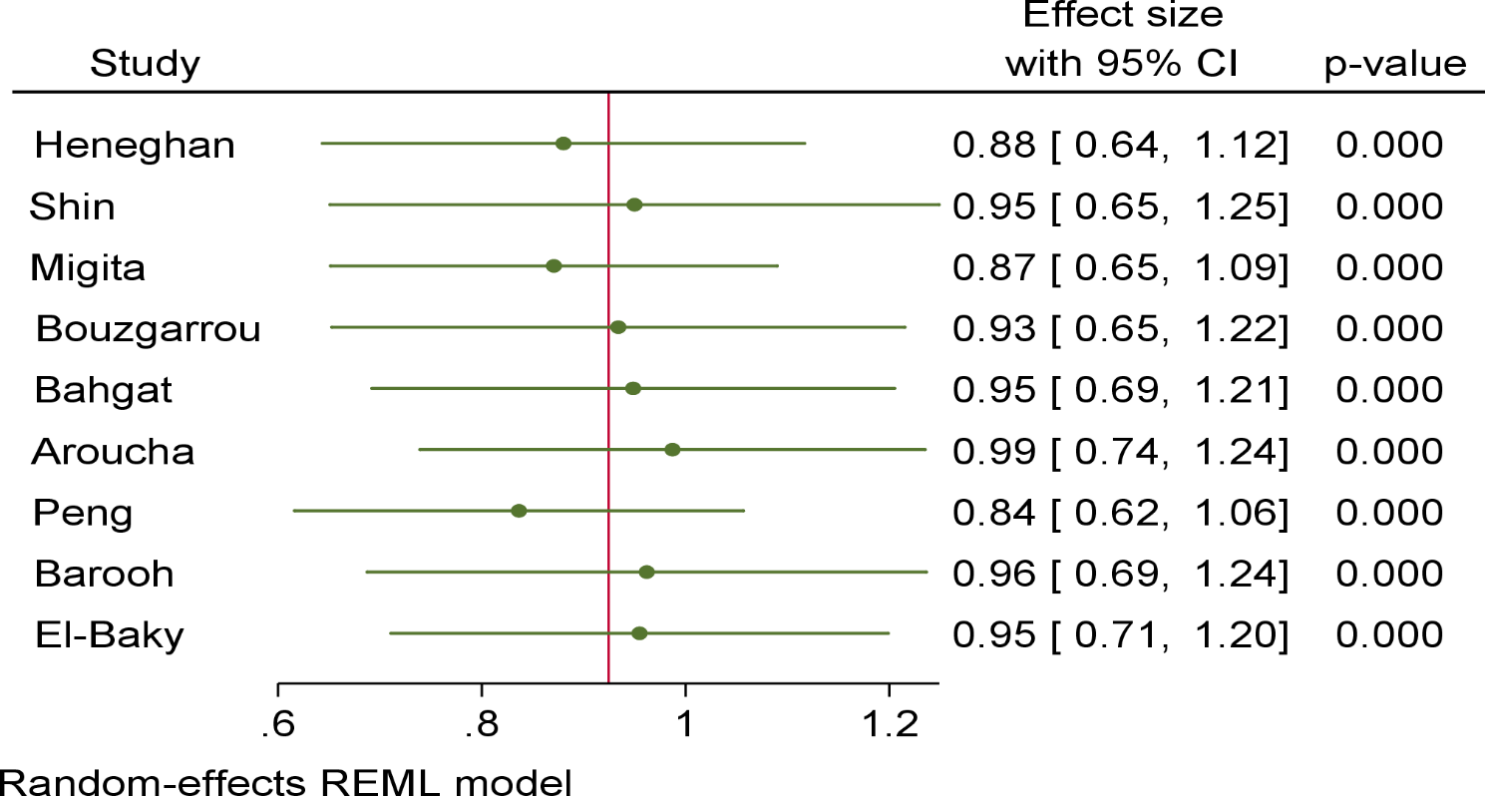
Forest plot of leave-one-out meta-analysis in IL-10-1082 A/G under dominant

For robustness of the findings, we performed’ leave-one-out meta-analysis’ by sequentially removing each study. For the SNP − 1082 A/G (nine studies), the omission of any single study did not significantly change the direction of estimates (Fig. 4). This was also found for IL-10 **(**-819 T/C and − 592 A/C) under all five genetic models (data not shown). These implied the stability of the effect estimates.

### Publication bias

For the SNP (-592 A/C) (11 studies), publication bias was detected in the dominant model (*p* = 0.051), homozygous (*p* = 0.059), heterozygous (*p* = 0.066), and allelic (*p* = 0.050) models. Due to less than ten studies included, we could not investigate publication bias for IL-10 **(**-1082 A/G) and IL-10 **(** -819 T/C).

### Trial sequential analysis

**Fig. 5:**
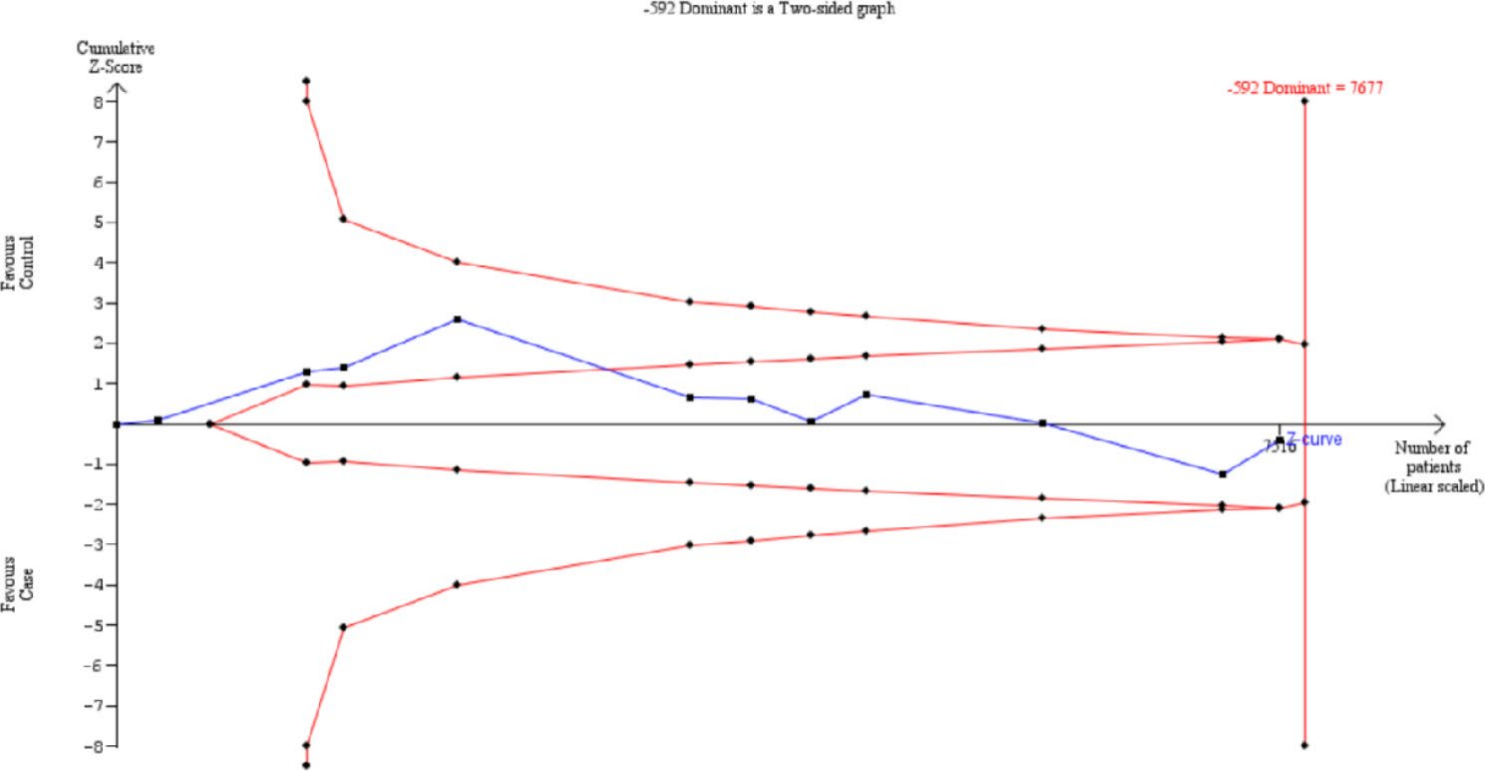
Trial sequential analysis plot of IL-10 (-592 A/C)

TSA approach was performed for all three SNPs using an overall 5% type I error and 80% power. For IL-10 **(**-592 A/C), the cumulative z-curve cut crossed the futility boundary, indicating the information size was adequate to establish a firm conclusion (Fig. 5).

In IL-10 -1082 A/G and − 819 T/C, the cumulative Z-curves did not pass the monitoring thresholds and was not within the futility area, indicating that the existing sample size for these SNPs were not adequate to establish a firm conclusion. This implied that the available information size in these two SNP (-1082 A/G and − 819 T/C) were not sufficient to provide firm evidence on the relationship with hepatocellular carcinoma. Therefore, further case-control studies investigating with IL-10 -1082 A/G and − 819 T/C are needed to provide a more robust conclusion.

## Discussion

The candidate gene approach is increasingly attractive to distinguish susceptibility genes that may trigger the initiation and progression of various types of cancer. In the present meta-analysis, we assessed the role of three important SNPs in the IL-10 gene (-1082 A/G, -819 T/C and − 592 A/C) in the risk of hepatocellular carcinoma across eight countries.

The gene encoding IL-10 is located on chromosome 1q31-1q32, and has three common IL-10 promoter variants (-1082 A/G, -819 T/C, -592 A/C) that significantly affect the gene transcription and expression [49]. Cytokines play an important role on the pathogenesis of virus-associated hepatitis. Clearance of hepatitis viruses following acute infection is associated with a vigorous cytotoxic T-cell response, through inhibition of viral replication and gene expression by the proinflammatory and Th1 cytokines [46, 50]. IL-10 is a potent suppressor of proinflammatory, and Th1 cytokine production [50], subsequently attenuate these inflammatory responses and reduce liver injury [46].

Our findings suggest IL-10 pertinent to AA and GG genotypes in -1082 A/G would be a protective role in hepatocellular carcinoma. A published study reported that high levels of IL-10 reduced the risk of hepatocellular carcinoma by reducing hepatic inflammation, which in turn prevents the malignant transformation of liver cells [51]. The significant association with subgroup of the non-Asian population implied that even the same polymorphism, there might have different effects on individuals from different ethnic backgrounds. Ethnicity plays a role in giving the genetic diversities among two different ethnic groups, leading to different susceptibilities to hepatocellular carcinoma [52]. With regards to -819 T/C, there was significant association with hepatocellular carcinoma under dominant model in the non-Asian population. This might be due to a prominent role of TT homozygous and CT heterozygous genotypes, which were linked to hepatocellular carcinoma. Among different ethnicities, the same gene polymorphism may influence differently among various ethnic populations. There were only four studies in IL-10 (-1082 A/G) and two studies in IL-10 (-819 T/C) for the non-Asian group. This reflects that non-Asian populations do not share the common genetic basis. A large-scale study in non-Asian populations is thereby require to substantiate the current findings.

It has been established that hepatocellular carcinoma is a multifactorial process, involving genetic factors and other factors such as lifestyle and environment factors, for the carcinogenesis [12]. Therefore, variations in lifestyle factors such as alcohol consumption and diet intake among participants in these ethnic groups may also contribute to the different susceptibilities to hepatocellular carcinoma even for the same polymorphism [53].

For IL-10 (-592 A/C), no significant association in both Asians and non-Asians might be explored that IL-10 − 592 A/C had neither the causal nor inhibitory effect in hepatocellular carcinoma development in these study population. Preclinical studies reported that − 592 A/C did not play a part in altering the level of production of IL-10 [54, 55].

### Comparisons with published reviews

A review including ten studies [56], assessed only allelic and dominant models, and reported no significant associations with any of these three SNPs and hepatocellular carcinoma. Another published review including 12 observational studies [21] reported a marginal association between IL-10 (-1082 A/G) and hepatocellular carcinoma for overall population, while significant association with − 592 A/C under dominant and allelic models for overall population, but no significant association with − 819 T/C under any genetic models for overall population or subgroups. IL-10 (-1082 A/G) and hepatocellular carcinoma were significantly associated in the non-Asian population under three genetic models (dominant, heterozygous, and allelic models), and IL-10 (-819 T/C) was significantly associated in the non-Asian population under three genetic models (dominant, recessive, and homozygous models), according to our review of 15 studies that evaluated five genetic models. However, TSA indicated that the significant relationships reported in our review may be spurious effects due to inadequate information (sample) size. Another review including seven case-control studies reported no significant associations between IL10 (-819 T/C) and hepatocellular carcinoma risk [20]. The published reviews did not assess adequate information size using the TSA method. This review applied TSA for the required information size, which is important to classify the effect estimates as ‘firm evidence of effect’ or ‘potentially spurious evidence of effect’ [30, 31]. Based on the TSA report, more sample size was required for the assessment of -1082 A/G and − 819 T/C in hepatocellular carcinoma risk for confirmatory evidence.

The difference in the number of included studies along with different total participants between the published reviews [20, 21, 56] and the current study may be the reason of the discrepancy in the findings. With SNPs (-819 T/C, -592 A/C) that had fewer studies than the recommended minimum limit of ten studies [26], the Zhang review [21] still performed publication bias. The current assessment, which is supported by TSA plots, as well as the previous reviews [20, 21, 56], concurred that findings should be interpreted with caution.

### Study limitations

There are several limitations that should be acknowledged. First, the sample sizes in the current study were small. For example, less than ten studies were recruited in the overall meta-analyses of -1082 A/G and − 819 T/C, and only two studies included for subgroup analysis of -819 T/C. Hence, there may be a type II error, regarding the small number of studies.

It is possible for specific environmental and lifestyle factors to alter those associations between gene polymorphisms and hepatocellular carcinoma risk [21]. However, we could not assess adjusted estimates with these potential confounding variables due to a lack of data or inconsistent reporting of these data. TtThe FRPP, which is the probability of no association between a genetic variant and disease (hepatocellular carcinoma in this case) given a statistically significant results, depends on the observed *p* value, the prior probability that the association between the genetic variant and the disease is real, and the statistical power of the test [28,57]. In this analysis, the FPRP approach did not support the associations as noteworthy of true associations at the 0.001 level. The FPRP approach is essentially Bayesian in that it formally integrates data from direct observation of study results with other information about the likelihood of a true association [28]. TSA plots also demonstrated the issue of inadequate samples in the two SNPs (-1082 A/G and − 819 T/C). In some studies, the genotypic distribution of controls did not conform to the HWE. Nevertheless, sensitivity analysis showed the stability of the estimates. Moreover, we included only published studies in English. Hence, there might be relevant studies in other languages or non-published studies, which could contribute to selection bias. Lastly, due to the complexity of the etiology of hepatocellular carcinoma, there might be a contextual interaction between polymorphisms of IL-10 gene and other genes together with environmental/lifestyle related factors, which were beyond the scope of our study [5–60]. Taken together, the current findings are limited guidance for screening susceptible populations in the real world.

Nevertheless, the current meta-analysis has strengths. We could identify a greater number of case- control studies that could enhance the power of the current meta-analysis, and additional TSA plot. The earlier reviews did not address adequate information size using the TSA method. The present study performed TSA as the required information size is important to classify the effect estimates as ‘firm evidence of effect’ or ‘potentially spurious evidence of effect’ [30, 31].

### Implications for clinical practice

Based on limited data presented in this review, we still do not know whether IL10 gene polymorphisms (-1082 A/G and − 819 T/C) increases or reduces the risk of hepatocellular carcinoma. Our analysis highlights the need for additional studies on the role of the IL10 gene in the development of hepatocellular carcinoma, and the findings should be used to inform healthcare providers considering screening of genetic risk factors. Such research could aid in identifying patients who have considerably higher risks of disease progression and may guide the development of customized treatment plans for chronic HCV infection. Since IL10 can diminishes the antiviral response, a practical approach could be the indication of earlier HCV treatment for those carrying low expression of IL-10. This would halt the inflammation induced by HCV infection and potentially inhibit the development of hepatocellular carcinoma [34].

## Conclusion

The findings suggested that IL-10 -1082 A/G and − 819 T/C have some roles in associated risk of hepatocellular carcinoma in the non-Asians group. The information size for confirmatory evidence was adequate only in the IL-10 (-592 A/C) to reach a conclusion. Future well-designed case-control studies with adequate number of participants in multi-ethnic groups are recommended to substantiate the evidence on the relationship between these two polymorphisms (IL10- 1082 A/G and − 819 T/C) and hepatocellular carcinoma.

### Electronic supplementary material

Below is the link to the electronic supplementary material.

Supplementary Material 1: PRISMA 2020 Checklist

Supplementary Material 2: Search strategies in databases

Supplementary Material 3: Generic formula

Supplementary Material 4: Excluded nine studies

Supplementary Material 5: Frequency of genetic polymorphisms

Supplementary Material 6: Methodological quality of studies assessed via NOS criteria

Supplementary Material 7: Forest plot of ? 819 C/T (a) dominant model (b) heterozygous model

## Data Availability

All data generated or analyzed during this study are included in this published article and its supplementary information files.

## List of abbreviations

CI: Confidence interval
FPRR: False positive report probability
HWE: Hardy–Weinberg equilibrium
IL-10: Interleukin-10
MAF: Minimum Alley frequency
NOS: Newcastle–Ottawa scale
OR: Odds ratio
PCR: Polymerase chain reaction
PCR/RFLP: PCR/restriction fragment length polymorphism
PCR-SSCP: Polymerase chain reaction-single-strand conformation polymorphism
PRISMA: Preferred Reporting Items for Systematic reviews and Meta-Analyses
RT-PCR: Reverse transcription polymerase chain reaction
SNP: Single nucleotide polymorphisms
