# Supplementary figures and images for: Association between IL-10 gene polymorphisms (− 1082 A/G, -819 T/C, -592 A/C) and hepatocellular carcinoma: a meta-analysis and trial sequential analysis
Source: BMC Cancer. 2023 Sep 8;23:842. doi: 10.1186/s12885-023-11323-1 (PMC10492326; doi:10.1186/s12885-023-11323-1)

**Additional File 2 – Search strategies in databases**


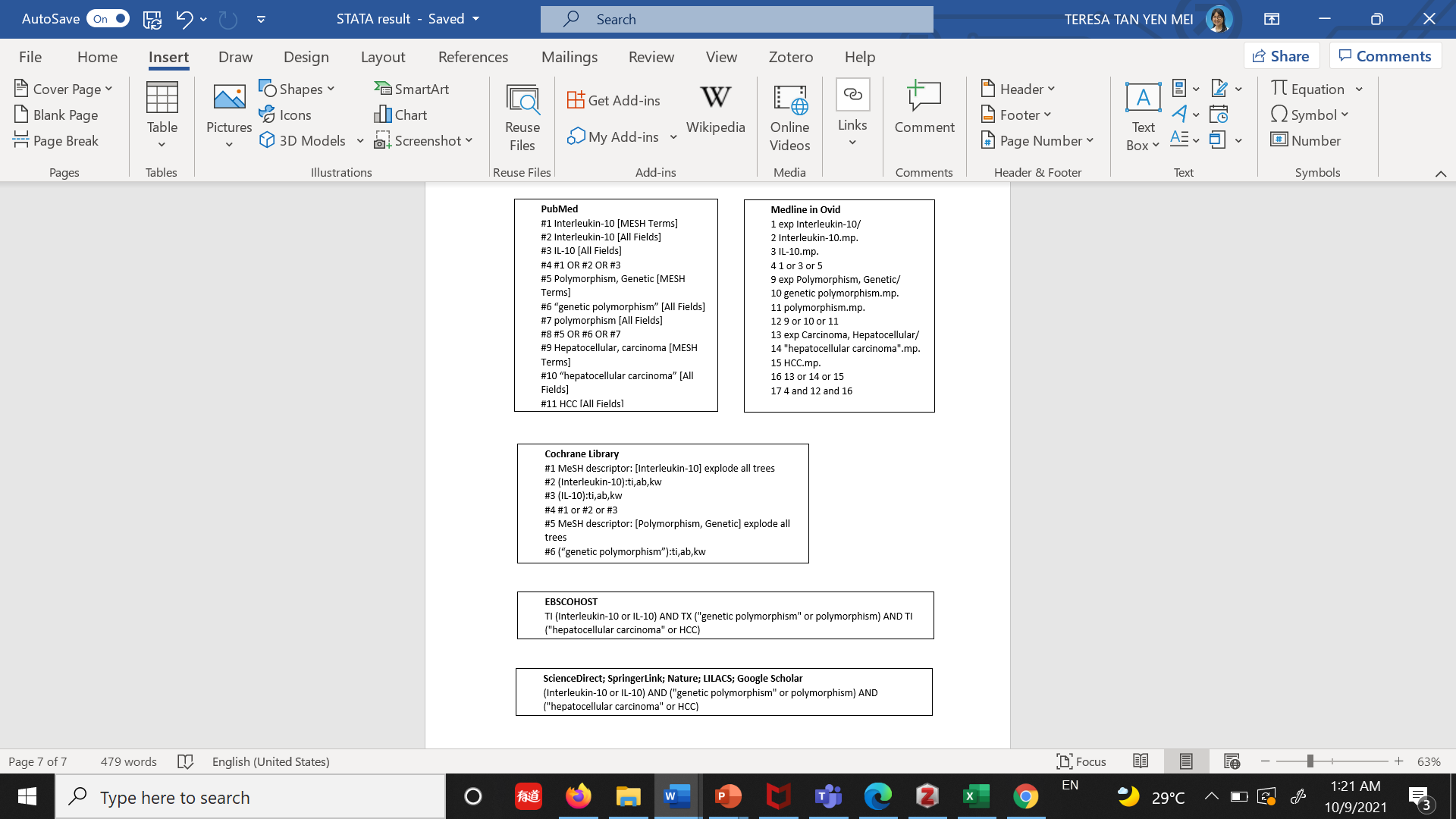

Supplement: Supplementary file 2 — Supplementary Material 2: Search strategies in databases [file 12885_2023_11323_MOESM2_ESM.doc]

**Additional File 4. Excluded nine studies**


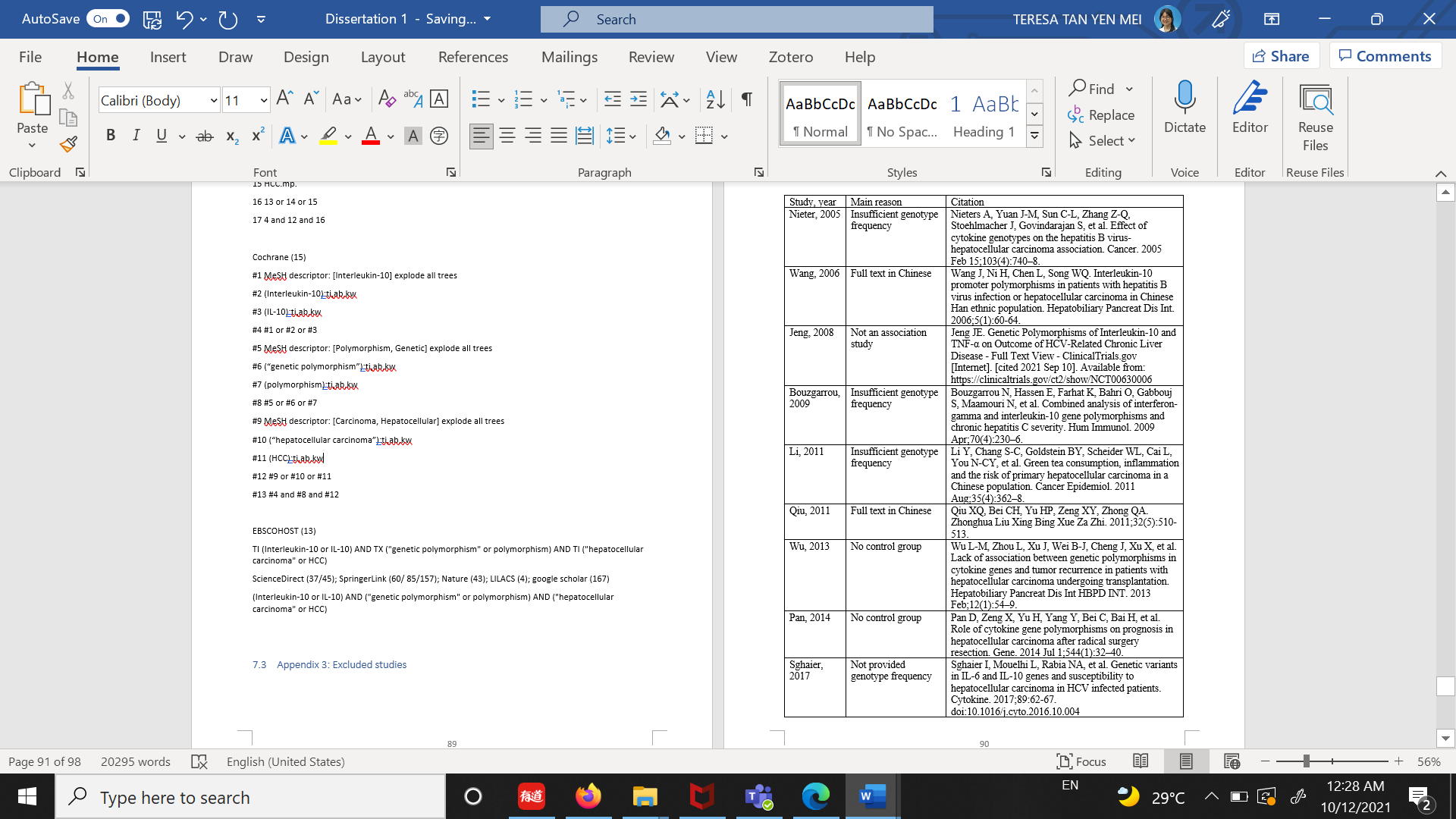

Supplement: Supplementary file 4 — Supplementary Material 4: Excluded nine studies [file 12885_2023_11323_MOESM4_ESM.doc]
