# Supplementary material for: Association between IL-10 gene polymorphisms (− 1082 A/G, -819 T/C, -592 A/C) and hepatocellular carcinoma: a meta-analysis and trial sequential analysis
Source: BMC Cancer. 2023 Sep 8;23:842. doi: 10.1186/s12885-023-11323-1 (PMC10492326; doi:10.1186/s12885-023-11323-1)
Supplement: Supplementary file 3 — Supplementary Material 3: Generic formula [file 12885_2023_11323_MOESM3_ESM.doc]

**Additional File 3. Generic formula**

**IL10 (-1082 A/G)**

- dominant (AG+GG vs AA),
- recessive (GG vs AG+AA),
- homozygous (GG vs AA),
- heterozygous (AG vs AA) and
- allelic (G vs A) models

**-819 T/C**

- dominant (CT+TT vs CC),
- recessive (TT vs CT+CC),
- homozygous (TT vs CC),
- heterozygous (CT vs CC)
- allelic (T vs C) genetic models

**-592 A/C**

- dominant (AC+AA vs CC),
- recessive (AA vs AC+CC),
- homozygous model (AA vs CC),
- heterozygous (AC vs CC)
- allele (A vs C) models.
