# Supplementary material for: Association between IL-10 gene polymorphisms (− 1082 A/G, -819 T/C, -592 A/C) and hepatocellular carcinoma: a meta-analysis and trial sequential analysis
Source: BMC Cancer. 2023 Sep 8;23:842. doi: 10.1186/s12885-023-11323-1 (PMC10492326; doi:10.1186/s12885-023-11323-1)
Supplement: Supplementary file 5 — Supplementary Material 5: Frequency of genetic polymorphisms [file 12885_2023_11323_MOESM5_ESM.doc]

**Additional File 5. Frequency of genetic polymorphisms**

| **Study, yr** | **Ref No.** | **Country** | **Case Total** | **AA** | **AG** | **GG** | **A** | **G** | **Control Total** | **AA** | **AG** | **GG** | **A** | **G** | **HWE** | **MAF** |
| --- | --- | --- | --- | --- | --- | --- | --- | --- | --- | --- | --- | --- | --- | --- | --- | --- |
| Heneghan,2003 | 40 | China | 98 | 86 | 12 | 0 | 184 | 12 | 175 | 160 | 15 | 0 | 335 | 15 | 0.5536 | 0.042 |
| Shin,2003 | 45 | Korea | 230 | 201 | 28 | 1 | 430 | 30 | 792 | 675 | 112 | 5 | 1462 | 122 | 0.8801 | 0.077 |
| Migita.2005 | 41 | Japan | 48 | 42 | 5 | 1 | 89 | 7 | 188 | 176 | 10 | 2 | 362 | 14 | 0.0004 | 0.37 |
| Bouzgarrou ,2009 | 38 | Tunisia | 58 | 24 | 24 | 10 | 72 | 44 | 145 | 56 | 68 | 21 | 180 | 110 | 0.9612 | 0.38 |
| Bahgat,2015 | 35 | Egypt | 50 | 16 | 26 | 8 | 58 | 42 | 50 | 11 | 28 | 11 | 50 | 50 | 0.3961 | 0.5 |
| Aroucha,2016 | 34 | Brazil | 108 | 61 | 37 | 10 | 159 | 57 | 280 | 125 | 122 | 33 | 372 | 188 | 0.699 | 0.33 |
| Peng,2016 | 42 | China | 173 | 83 | 74 | 16 | 240 | 106 | 182 | 96 | 74 | 12 | 266 | 98 | 0.6533 | 0.27 |
| Barooh,2020 | 36 | India | 60 | 33 | 15 | 12 | 81 | 39 | 306 | 141 | 114 | 51 | 396 | 216 | 0.0012 | 0.35 |
| El-Baky ,2020 | 39 | Egypt | 54 | 11 | 38 | 5 | 60 | 48 | 92 | 5 | 83 | 4 | 93 | 91 | 0.0000 | 0.49 |

HWE: Hardy-Weinberg equilibrium‘;MAF: minor alley frequency; yr: Year.

**IL10 -819 C/T**

| **Study,yr** | **Ref no.** | **Country** | **Case Total** | **CC** | **CT** | **TT** | **T** | **C** | **Control total** | **CC** | **CT** | **TT** | **T** | **C** | **HWE** | **MAF** |
| --- | --- | --- | --- | --- | --- | --- | --- | --- | --- | --- | --- | --- | --- | --- | --- | --- |
| Barooh,2020 | 36 | India | 60 | 13 | 23 | 24 | 71 | 49 | 306 | 60 | 139 | 107 | 353 | 259 | 0.2237 | 0.42 |
| Heneghan,2003 | 40 | Korea | 98 | 11 | 38 | 49 | 136 | 60 | 174 | 19 | 60 | 95 | 250 | 98 | 0.0513 | 0.28 |
| Migita,2003 | 41 | Japan | 48 | 8 | 23 | 17 | 57 | 39 | 188 | 25 | 78 | 85 | 248 | 128 | 0.2967 | 0.34 |
| Saxena,2014 | 44 | India | 59 | 9 | 47 | 3 | 53 | 65 | 331 | 52 | 248 | 31 | 310 | 352 | 0.0000 | 0.53 |
| Peng,2016 | 42 | China | 173 | 22 | 77 | 74 | 225 | 121 | 181 | 17 | 78 | 86 | 250 | 112 | 0.9097 | 0.31 |
| Wang,2019 | 47 | China | 554 | 182 | 304 | 68 | 440 | 668 | 612 | 250 | 306 | 56 | 418 | 806 | 0.00572 | 0.66 |

**IL10 -592 A/C**

| **Study,yr** | **Ref no.** | **Country** | **Case Total** | **CC1** | **A1** | **C1** | **Control total** | **AA** | **AC** | **CC** | **A** | **C** | **HWE** | **MAF** |
| --- | --- | --- | --- | --- | --- | --- | --- | --- | --- | --- | --- | --- | --- | --- |
| Shin,2003 | 45 | Korea | 216 | 26 | 279 | 153 | 748 | 384 | 299 | 65 | 1067 | 429 | 0.5328 | 0.29 |
| Migita,2003 | 41 | Japan | 48 | 8 | 57 | 39 | 188 | 85 | 78 | 25 | 248 | 128 | 0.2967 | 0.34 |
| Tseng ,2006 | 46 | Taiwan | 208 | 31 | 270 | 146 | 528 | 259 | 223 | 46 | 741 | 315 | 0.8383 | 0.3 |
| Barooh | 36 | India | 60 | 44 | 21 | 99 | 306 | 58 | 95 | 153 | 211 | 401 | 0.0000 | 0.65 |
| Bei,2014 | 38 | China | 720 | 52 | 1024 | 416 | 784 | 392 | 313 | 79 | 1097 | 471 | 0.1604 | 0.3 |
| Heneghan | 40 | Korea | 98 | 11 | 136 | 60 | 174 | 95 | 60 | 19 | 250 | 98 | 0.0513 | 0.28 |
| Saxena,2014 | 44 | India | 59 | 9 | 53 | 65 | 331 | 31 | 248 | 52 | 310 | 352 | 0.0000 | 0.53 |
| Aroucha | 34 | Brazil | 108 | 43 | 81 | 135 | 280 | 30 | 121 | 129 | 181 | 379 | 0.8378 | 0.68 |
| Peng,2016 | 42 | China | 173 | 35 | 195 | 151 | 182 | 79 | 81 | 22 | 239 | 125 | 0.8599 | 0.34 |
| Zhou,2017 | 48 | China | 430 | 35 | 617 | 243 | 709 | 291 | 340 | 78 | 922 | 496 | 0.1486 | 0.35 |
| Wang,2019 | 47 | China | 554 | 160 | 506 | 602 | 612 | 110 | 288 | 214 | 508 | 716 | 0.4456 | 0.58 |
| Migita | 2003 | Japan | 48 | 8 | 57 | 39 | 188 | 85 | 78 | 25 | 248 | 128 | 0.2967 | 0.34 |
