# Supplementary material for: Association between IL-10 gene polymorphisms (− 1082 A/G, -819 T/C, -592 A/C) and hepatocellular carcinoma: a meta-analysis and trial sequential analysis
Source: BMC Cancer. 2023 Sep 8;23:842. doi: 10.1186/s12885-023-11323-1 (PMC10492326; doi:10.1186/s12885-023-11323-1)
Supplement: Supplementary file 6 — Supplementary Material 6: Methodological quality of studies assessed via NOS criteria [file 12885_2023_11323_MOESM6_ESM.doc]

**Additional File 6. Methodological quality of studies assessed via NOS criteria**

| **Author, yr**  **[ref]** | **Domains** | | | **Total scores** |
| --- | --- | --- | --- | --- |
| Author, yr  [ref] | Selection | Compatibility | Exposures |  |
| Aroucha 2016  [34] | 2 | 2 | 2 | 6 |
| Bahgat 2015 [35] | 2 | 2 | 2 | 6 |
| Barooh 2020 [36] | 2 | 2 | 2 | 6 |
| Bei 2014 [37] | 2 | 2 | 2 | 6 |
| Bouzgarrou 2009 [38] | 3 | 2 | 2 | 7 |
| El-Baky 2020 [39] | 3 | 2 | 2 | 7 |
| Heneghan 2003  [40] | 3 | 2 | 2 | 7 |
| Migita 2003 [41] | 3 | 2 | 2 | 7 |
| Peng 2016 [42] | 1 | 2 | 2 | 5 |
| Saleh 2020 [43] | 2 | 2 | 2 | 6 |
| Saxena 2014 [44] | 3 | 2 | 2 | 7 |
| Shin 2003 [45] | 2 | 1 | 2 | 5 |
| Tseng 2006 [46] | 2 | 1 | 2 | 5 |
| Wang 2019 [47] | 3 | 2 | 2 | 7 |
| Zhou 2017 [48] | 2 | 2 | 2 | 6 |
