# Supplementary material for: Association between IL-10 gene polymorphisms (− 1082 A/G, -819 T/C, -592 A/C) and hepatocellular carcinoma: a meta-analysis and trial sequential analysis
Source: BMC Cancer. 2023 Sep 8;23:842. doi: 10.1186/s12885-023-11323-1 (PMC10492326; doi:10.1186/s12885-023-11323-1)
Supplement: Supplementary file 7 — Supplementary Material 7: Forest plot of ? 819 C/T (a) dominant model (b) heterozygous model [file 12885_2023_11323_MOESM7_ESM.doc]

**Additional File 7. Forest plot of − 819 C/T (a) dominant model (b) heterozygous model**
